# Supplementary material for: Training Scientific Communication Skills on Medical Imaging within the Virtual World Second Life: Perception of Biomedical Engineering Students
Source: Int J Environ Res Public Health. 2023 Jan 17;20(3):1697. doi: 10.3390/ijerph20031697 (PMC9914803; doi:10.3390/ijerph20031697)
Supplement: Supplementary file 1 [file ijerph-20-01697-s001.zip › File S1 questionnaires-review.pdf]

Teaching experiences on medical imaging with biomedical engineering students in the virtual world Second Life: training scientific communication skills

S1 – Evaluation questionnaire

Pilot experience with students as presenters of educational contents (year 2015).
Training of students in delivering oral presentations (years 2016 and 2017)

Likert scale

Please complete the following questions, scoring from 1 to 5 (1: totally disagree, 5: totally agree)

1. The contents seemed adequate for your education
2. The contents were very difficult for your current level of knowledge
3. You found this initiative in Second Life interesting
4. The environment of the island seemed attractive to you
5. The Floating Auditorium was adequate to carry out the sessions
6. You knew Second Life before this experience
7. You managed Second Life with ease
8. The tasks of creating and managing your avatar were easy
9. Your computer allows run Second Life without problems
10. Your Internet allows run Second Life without problems
11. You are willing to participate in another experience in Second Life this year
12. You are willing to participate in another experience in Second Life in next years
13. The intervention of the teacher was adequate
14. Session scheduling in Second Life was interesting
15. Your participation in Second Life was very active
16. Contact with your classmates in Second Life is good for learning
17. Doing a first web page presentation session is a good idea *
18. The format of the presentation of topics is very interesting
19. The selection of topics for discussion was adequate
20. The classmates who presented topics did it very well
21. Your participation in the debates was very active
22. The number of sessions was excessive
23. The number of sessions was insufficient

Overall rating

Please rate the following aspects, scoring from 1 to 10

1. The overall experience
2. The organization of the project
3. The educational contents
4. The utility for your education
5. The island environment in Second Life
6. The teacher in Second Life
7. The interaction with your classmates in Second Life
8. The sessions in Second Life
9. The connectivity in Second Life

Open comments

If you want to add something, please use the box below

* This statement was included only in 2016 and 2017 questionnaires
