# Supplementary material for: Training Scientific Communication Skills on Medical Imaging within the Virtual World Second Life: Perception of Biomedical Engineering Students
Source: Int J Environ Res Public Health. 2023 Jan 17;20(3):1697. doi: 10.3390/ijerph20031697 (PMC9914803; doi:10.3390/ijerph20031697)
Supplement: Supplementary file 1 [file ijerph-20-01697-s001.zip › File S2 opencomments-review.pdf]

Open comments 2015

Questionnaire 01: I find that using Second Life was a good way to make the course more interactive and to learn things that might otherwise have been more difficult / tedious to learn. But while it seems like a good idea to me, the fact that it has to be online and that it depends so much on the connection or the type of computer it harmed me a lot, since I could not make proper use of the platform and I could not see the scenarios well, the other participants, etc., I do not know if because of my internet connection or because of another factor that I do not know. It is true that at least it did allow me to see the screen, for which I had no problem, but I did, for example, every time I had to change places or move around or, for example, to take a seat in the class that we were in since it did not allow me to see hardly any structure, only to hear the voices. If I could change something, perhaps I would change the duration of the sessions to shorten them a bit because there were times that they were a bit long.

Questionnaire 02: *No open comments.*

Questionnaire 03: *No open comments.*

Questionnaire 04: *No open comments.*

Questionnaire 05: *No open comments.*

Questionnaire 06: I found it to be a very enriching experience, in pursuit of our development in the informatics and healthcare field. It has been a novel activity, which I would not mind repeating, since it helps us to develop orally, allowing us to investigate everyday topics and establish interesting debates, leaving us out of the monotony of the conventional class.

Questionnaire 07: *No open comments.*

Questionnaire 08: I consider the introduction of an experience that allows interaction in this way to be interesting. However, I think the amount of time spent on these types of sessions is really excessive. I think it's very good that you try to present the students as much as possible when talking about the different topics (asking for their opinion and encouraging their participation). Although in general the experience is excessive, that is, it is good to have a little contact with the virtual world, but it has been too long.

Questionnaire 09: *No open comments.*

Questionnaire 10: I think that this activity is good to complement our training, since with the class hours we have and a single course in which they talk to us about obtaining and improving biomedical images, it is not possible to cover all the ways that exist to obtain different images. Thus, with the interventions of the classmates and the teacher, in the different sessions, we have been able to learn about the existence of other techniques.

Questionnaire 11: *No open comments.*

Questionnaire 12: *No open comments.*

Questionnaire 13: *No open comments.*

Questionnaire 14: *No open comments.*

S2 – Open comments

Questionnaire 15: *No open comments.*

Questionnaire 16: The experience with Secondlife in general has seemed very suitable for our training, a teaching method different from the one we are used to, comfortable and easy to use.

Questionnaire 17: *No open comments.*

Questionnaire 18: *No open comments.*

Questionnaire 19: *No open comments.*

Questionnaire 20: *No open comments.*

Questionnaire 21: *No open comments.*

Questionnaire 22: *No open comments.*

Questionnaire 23: *No open comments.*

Open comments 2016

Questionnaire 01: I think time could have been saved with an introductory class where everyone would have learned to use Second Life or if my classmates had previously tried the application on their own. It became tiring to have to spend a large percentage of the session time trying to solve problems at the level of use (Microphones, PDF on screen ...), not to mention the colleagues who were misusing and aware of the platform, although that can only be fixed with maturity on their part. There are things that, luckily or unfortunately, technology cannot remedy.

Questionnaire 02: *No open comments.*

Questionnaire 03: The experience in Second Life has been quite good, it is a very interesting environment for additional explanations and work exhibitions. I would very much like it to be used for other subjects or in future work.

Questionnaire 04: *No open comments.*

Questionnaire 05: The overall experience has been interesting, formative and very attractive. I only have one complaint-suggestion: this activity has required a lot of work on the part of the students. Some of us have had to move plans to be able to leave the afternoons free and thus be able to participate in the sessions. The presentation, although we have been able to do it from home and expose them with a script in front of it, must be prepared, check the operation in the Second Life environment (as we have verified, in some cases the slides were not passed correctly for everyone, in others there was no sound or videos), studying, looking for information ... The suggestion itself does not lie in the complexity of the work, but in the fact that I do not think that the percentage of this activity corresponds to the final grade (10%), given the time that should be devoted. Perhaps for future years it would be better to increase the percentage while maintaining the difficulty? Or that, if your group has a grade higher than the average of all the groups, this part of the subject (Second Life) counts twice (20%), for example. Even so, thank you very much for placing confidence in the students to carry out these sessions. Few teachers are capable of innovating in this way.

Questionnaire 06: *No open comments.*

Questionnaire 07: The idea of Second Life is original and different. It also helps us not to pass that fear during the expositions to the rest of the classmates, but a presentation could also be made in class to confront that "fear". For the rest, a very interesting content about the world of biomedical images that I would not have time to see in class, although sometimes it costs a little to keep the attention on the topic or the discussion due to the failures with the audio or the connection and due to the fact of not having the participants present.

Questionnaire 08: Personally, I am very happy to have participated in the Second Life activity. It seems to me a great way to interact with both the teacher and the classmates, it is also very comfortable since it can be done from home. I hope this is not my last Second Life experience.

Questionnaire 09: I think that in terms of content it is very suitable for the subject, however, in my opinion sessions should be divided with Second Life and face-to-face, where in the latter they take a more practical and applied field as far as possible directly to engineering.

Questionnaire 10: *No open comments.*

Questionnaire 11: I see more important that, instead of using this tool to cover more content, which is given in a shallow way, to use those hours to go even deeper into the topics that are supposed to go into the exam. Mainly, because I think that it is better to know a lot about something specific, than a little about many things. However, the organization and the achievement of the subject seemed optimal to me. It was just commenting on that personal opinion. Thank you for your time and attention for the course.

Questionnaire 12: *No open comments.*

Questionnaire 13: I have found it an interesting environment and the topics we have worked on have seemed very formative. But what has seemed a little worse compared to a normal presentation in class has been the fact that some presentations did not work well and that all the classmates could handle the screen since they have been able to manipulate some presentation, harming the people who were exhibiting at that time.

Questionnaire 14: I think that the group presentations should not have been evaluated in their entirety by the classmates, I think that half of that note should be the teacher's evaluation, since, depending on the extracurricular relationships of the groups, they can make the evaluation of these exhibitions be subjective and not objective as sought.

Questionnaire 15: I believe that the sessions would be better scheduled in the morning in the free classes that we have not used since there are people who have other activities or studies organized in the afternoons and interrupt them. Otherwise I think it has been a rewarding and innovative experience that serves to learn in a more playful way.

Questionnaire 16: *No open comments.*

Questionnaire 17: *No open comments.*

Questionnaire 18: *No open comments.*

Questionnaire 19: I have found it to be a very educational and useful resource.

Questionnaire 20: I have found it interesting to work with Second Life. I'm not very into virtual and role-playing games and it was good to try something different. However, I think it is easier to make an exhibition through a screen (and behind an avatar) than in person and I suppose that face to face serves us more as an experience for the future. I also would have liked the topics in the exhibitions to be a bit broader since almost all of them were about x-rays and it might also have been interesting to learn about other imaging techniques like the others that you proposed. All in all, thank you very much for the sessions and your interest in us, you are both very good teachers.

Questionnaire 21: Using Second Life to continue the course has been interesting. However, I would have preferred to give these sessions in person in class, thus saving the problems with the schedule, installation of the program and the problems that arose during the sessions with the computer, whether it was audio, microphone, etc.

Questionnaire 22: *No open comments.*

Questionnaire 23: It seemed like a good experience to me, but the way we evaluated the exhibitions has not been very objective on our own part. I think it is an innovative method and that it can be attractive to teach. The main problem that I see is that it is very easy to get distracted, since we are in front of a laptop, with an Internet connection and nobody is watching us.

Questionnaire 24: *No open comments.*

Questionnaire 25: *No open comments.*

Questionnaire 26: *No open comments.*

Questionnaire 27: I would have liked that some colleagues had taken it a little more seriously, leaving aside some jokes and other types of events that sometimes impeded the advancement of certain groups when presenting their topics.

Questionnaire 28: *No open comments.*

Questionnaire 29: From my point of view, in our career familiarization with new technologies is vital, almost survival, so it has not been difficult to get used to the routine followed in these sessions. This takes a point in favor, because no time is wasted in learning to manage the environment and you can go directly to what is important, which are the training activities. It is always positive to take extra hours to review even expand knowledge of a subject that should be so familiar to us in our field, that is why I qualify this experience as something productive. In addition, being something different helps to awaken interest in the student. If I could define this experience in some summarized way, I would say that it is like going to class but from home. All the comforts of being at home along with the advantage of going to class outside of regular hours to review or expand on the syllabus. Something like that can never be negative.

Questionnaire 30: *No open comments.*

Questionnaire 31: *No open comments.*

Questionnaire 32: *No open comments.*

Questionnaire 33: Second Life seems interesting to me as a concept, but that the sessions are in the afternoon and of such a long duration does not seem so correct. Let me explain, I usually have activities in the afternoons, like English or sports, and having to cancel those activities (which I'm paying for) in order to go to Second Life sessions bothers me a bit. My solution to this problem would be to hold Second Life sessions on a weekend and how this is to agree students and teacher, then decide well in advance the days to organize the sessions so that we as students can better organize with the rest. of activities that we carry out outside the university. What I mean is to do the sessions at a time that everyone can attend without having to suspend extracurricular activities. If for us it is an effort of time, it is also for the teacher and I wanted to thank the teacher for his work, giving us his time and organizing these types of activities that are out of the ordinary, which are often appreciated. Greetings and thank you very much.

Questionnaire 34: *No open comments.*

Questionnaire 35: I found it very interesting, novel and the topics to be discussed very much in line with the subject.

Questionnaire 36: I think that the assessment of my group is unfair and does not reflect the work done.

Questionnaire 37: *No open comments.*

Questionnaire 38: It is novel, but in some sessions it became a bit tedious.

Open comments 2017

Questionnaire 01: *No open comments.*

Questionnaire 02: *No open comments.*

Questionnaire 03: It seems to me a totally different activity than what we are used to, for that reason I liked it, the only drawback is that sometimes the computer or the speed of the Internet prevent you from enjoying the platform more. Another drawback is the schedule, since it is complicated those weeks in which we had practices of the syllabus apart from those of Second Life, also adding practices of other subjects. Otherwise without problems, since the sessions were entertaining, especially the debates that took place as a result of the exhibitions.

Questionnaire 04: I found it very interesting and entertaining, but you need very good computers and internet connections, and it is difficult to have both. It would be nice to be able to connect from the university's Wi-Fi since it is not possible.

Questionnaire 05: *No open comments.*

Questionnaire 06: *No open comments.*

Questionnaire 07: *No open comments.*

Questionnaire 08: It seemed to me an interesting experience but, although given the content proposed to develop it could not be less, I think it was an excessive amount of time that could lead to disconnection on our part. Perhaps another way of dividing the sessions, shorter, or even fewer activities could have given a greater productivity in these. I also think that I would avoid this 'disconnection' by doing more group activities where everyone present can participate together at the same time and not only at a predetermined time for it such as the corresponding exposure of their group. For the rest, I think it is a very interesting environment that could be used a lot.

Questionnaire 09: *No open comments.*

Questionnaire 10: In addition to the various technical problems that there were, from problems with the audio and microphones to broken graphics cards, I think the Second Life sessions were adequate. The real problem is not the number of sessions, but the length of time they last. In my opinion, several sessions of 1 hour would be more productive than 2 hours, since two hours watching people talk about class topics in the afternoons is quite tiring and the last half hour is very disconnected and not attentive.

Questionnaire 11: *No open comments.*

Questionnaire 12: *No open comments.*

Questionnaire 13: *No open comments.*

Questionnaire 14: It seemed like a different and interesting way of learning, but from my point of view, the organization has not been entirely correct on some occasions, mainly due to the loss of time caused by some incidents. In general, I think it is a good initiative that if we improve more it could give very good results.

Questionnaire 15: *No open comments.*

Questionnaire 16: The experience in Second Life has seemed quite positive to me, learning different ways of presenting a work with the advantage of not having to do it in person and that everyone can attend from "anywhere". The teacher's work seemed adequate to me. The topics covered in general have seemed correct to me, as well as the work done by my colleagues. Like almost everything, it has its drawbacks. Connectivity has been difficult for some colleagues at times. Although in my case my computer has had enough power to support the Second Life program without problems, nevertheless, there have been colleagues who have had difficulties with their equipment. (Like for example my exhibition partner, that the audio from his team did not work with the program and he had to do the joint exhibition from my computer). In general, a positive experience but with some technical difficulties that, in the coming years, should be solved for the proper development of the activity.

Questionnaire 17: *No open comments.*

Questionnaire 18: *No open comments.*

Questionnaire 19: *No open comments.*

Questionnaire 20: *No open comments.*

Questionnaire 21: *No open comments.*

Questionnaire 22: *No open comments.*

Questionnaire 23: The experience in Second Life in general has not been bad, but I consider that the time we have spent doing this has been excessive. Personally I prefer face-to-face classes.

Questionnaire 24: It has been interesting to know and try other ways of presenting topics related to the subject. But, in my case, these sessions have not taught me much. It is a technique in which you have to be concentrated during the two hours that it lasts and in many occasions you get lost because you do not hear well, the microphone does not work, the internet connectivity fails, ... I have also seen too many hours that we have dedicated to this. Perhaps my opinion is more "classical" and I prefer to have taught these subjects in class. However, it has been one more new experience.

Questionnaire 25: *No open comments.*

Questionnaire 26: *No open comments.*

Questionnaire 27: *No open comments.*

Questionnaire 28: In my case it was a bad experience and not because of the activity itself, but because my computer did not have a suitable graphics card to support several sessions of the game. I already knew the limit of my computer but since it was a mandatory activity and with no option to do it on a computer at the university, I had no alternative but my computer and finally the graphics card to use broke. It is an expense that the university will not pay and I am the injured party. If everything had happened differently, it had been an interesting experience. I would suggest that it ceases to be a mandatory activity or that we have some option for the availability of computers at the faculty so that no one goes through my problem in the future. Since Second Life is quite a heavy game and more than one of my colleagues has had problems

with his computer as this game causes laptops to overheat. That being said I would like to thank you for your compression for my absence in parts of the sessions after breaking my graphics card.

Questionnaire 29: Second Life seemed fine to me just for the fact that I didn't need to go to class for the session, in fact I was ironing while they finished, but the subsequent discussions I don't think are adequate, many presentations seemed to me that they were not complete, and something bad, but I did not want to "offend" the colleagues with the presentation and I kept quiet. The evaluations have seemed good to me but the debate, except in some group, was to say things that have been done wrong or were not understood, I would prefer as a questionnaire about the presentations that are later answered by those who listen, or something like that, although it may be also not a good method.

Questionnaire 30: *No open comments.*

Questionnaire 31: I think that these types of activities could be replaced by others in which we interact better with our classmates or teachers. In addition, sometimes he did not know if he should ask some questions because he did not know if they had been explained and he had not been able to listen to him due to a connection problem. If he asked and it had been explained, it was interpreted as not having been attentive. I think that this activity has not contributed anything to me having done it through Second Life.

Questionnaire 32: I have found something very useful that in this subject it is presented as a rather complementary training. But the fact of even proposing classes in this way seems very interesting to me.

Questionnaire 33: It seems to me a very original way of connecting with the students. It is different from what other teachers propose, with also the ease of doing it even from home. It is also a good way to encourage the quietest and most timid to speak in public and express themselves as they have the advantage of "anonymity" and not seeing faces. Finally, the proposed topics are very interesting and allow a different approach to the subject (although some of them may seem repetitive, depending on the approach of the presenting group). However, there are several cons that I think should be taken into account. To begin with, and what has been most important to me, is that it is a very heavy program, needs a good internet connection and uses too many processes. In my case, this has resulted in black screen sessions because my computer couldn't handle it, which is not good. On the other hand, although the initiative gives the convenience of doing it from home, it is after class hours, and in the middle of the afternoon, which breaks the organization of the day. It is difficult to organize oneself around this, since we have to be quite attentive to the 2 hours that the exhibitions last. To finish, say that there is a group of people who on the first day of class at the presentation said that they had never spoken in public. This activity is very good as I have already said to encourage them to speak, but it could be better if they followed a class first by themselves to learn to communicate, since many times the differences in the quality of the presentations were too great. And we all know that a job that is badly exposed is a job that is rarely heard by other colleagues. In conclusion, I would say that it is a very good initiative, but that it could take place in a less heavy setting and during class time.

Questionnaire 34: I think the web page presentation class was unnecessary. Technical problems encountered by colleagues have been solved efficiently, without greatly interrupting the dynamics of the session.

Questionnaire 35: *No open comments.*

Questionnaire 36: I think that Second Life sessions can get a bit heavy and even turn out to be a waste of time (as they have been planned), besides it overheats the computer a lot, the nerves that I spend in the sessions are greater than if had to expose in class. A lot of time is wasted to properly start the session, sometimes the session ended half an hour later than planned. Yes, it is true that you learn very interesting things, but rather by way of curiosity, because then you forget them immediately, due to the emotional stress and the burden of undergoing the sessions after having had classes all morning. Perhaps it could be interesting if they were fewer sessions and of shorter duration, because that way we would not get so tired, more would be learned and the stress would be less.
